# The effect of baby touch on premature infants

**DOI:** 10.12669/pjms.40.10.9275

**Published:** 2024-11

**Authors:** Qiuying Du, Hongbin Zhu, Man Guo, Qingqing Yang, Huichan Yang, Yanfei Ma

**Affiliations:** 1 Qiuying Du, Department of Paediatrics, Maternity & Child Care Center of Qinhuangdao, Qinhuangdao 066000, Hebei, China; 2 Hongbin Zhu, Department of Paediatrics, Maternity & Child Care Center of Qinhuangdao, Qinhuangdao 066000, Hebei, China; 3 Man Guo, Department of Paediatrics, Maternity & Child Care Center of Qinhuangdao, Qinhuangdao 066000, Hebei, China; 4 Qingqing Yang, Department of Paediatrics, Maternity & Child Care Center of Qinhuangdao, Qinhuangdao 066000, Hebei, China; 5 Huichan Yang, Department of Paediatrics, Maternity & Child Care Center of Qinhuangdao, Qinhuangdao 066000, Hebei, China; 6 Yanfei Ma, Department of Paediatrics, Maternity & Child Care Center of Qinhuangdao, Qinhuangdao 066000, Hebei, China

**Keywords:** Touch, Premature infant, Growth and development, Immunological function, Sleep quality, Emotional communication

## Abstract

**Objective:**

Premature infants require special attention during nursing. This study aimed to explore the effects of baby touch on the growth, immunity, sleep and communication in premature infants.

**Methods:**

This was a retrospective study. A total of 64 premature infants with a gestational age of 30-36 weeks were selected at Maternity & Child Care Center of Qinhuangdao from June 2022 to May 2023, and randomly divided into experimental group and control group. The control group received routine comprehensive nursing care for newborns, while the experimental group was additionally treated with touch intervention using the international standard touch method. The effects of the three months touch on the growth and development, immunological function, sleep quality, and emotional communication of the premature infants were observed.

**Results:**

No statistically significant differences were found in gender, gestational age, birth weight, birth length, or head circumference at birth between the two groups (*p>* 0.05). In the third month, the increases in body weight, body length and head circumference were significantly more obvious, the immunological function indicators including CD^3+^, CD^4+^, CD^8+^, IgG, IgA and IgM were all significantly higher, the sleep time and sleep quality were both significantly superior, and the interaction time and intimacy with parents were significantly superior in the experimental group compared with the control group, with statistically significant differences (*p<* 0.05).

**Conclusion:**

Baby touch can promote growth and development, strengthen immunological function, improve sleep quality and enhance the emotional communication abilities of premature infants, and help them better adapt to the environment and grow.

## INTRODUCTION

The World Health Organization (WHO) defines premature birth as a birth before 37 completed weeks of gestation, or at a gestational age of less than 259 days from the last menstrual period,^1^ which may be an adverse pregnancy outcome (i.e., a fetus is unable to achieve its growth potential in the uterus) or a preferred outcome (e.g., a premature birth successfully avoids abortion or inability to survive). Even among healthy women with low-risk pregnancies, premature birth can still be predicted in some fetuses.^2^ Premature birth is a situation where the pregnancy fails to last for a certain period of time, rather than the appearance of specific signs or symptoms. Due to the premature birth of an infant, its physical development and physiological functions are not yet fully mature.^3^ For newborns, premature birth is a risk factor that affects their health, well-being and development into adulthood. Premature infants are more susceptible to illness than full-term infants, especially due to body temperature instability, hypoglycemia, respiratory distress, apnea, jaundice and feeding difficulties.^4^-^6^ Consequently, premature infants require special attention and care during nursing.

A study on the short- and long-term effects of adjustable fortification regimen on the growth parameters of premature infants in the neonatal intensive care units (NICUs) and after discharge has shown that targeted regimen and nursing can increase protein intake, further enhancing the physical growth rate of premature infants.^7^ Touch is a widely used nursing practice in recent years, which is important in various social aspects such as parent-child relationship and subjectivity. It is the first direct sense of maturity that an individual obtains from the uterus, and is the core of social contact and meaningful interaction. From a medical, developmental and psychological perspective, touch is crucial for the life and development of infants.^8^-^10^ Baby touch is a touch-based nursing method that can reduce stress and provide tactile stimulation. Therefore, it is recommended as an intervention to promote the growth and development of premature infants and low birth-weight infants and is believed to promote the physical and mental health and development of infants.^11^ It has been demonstrated that touch has significant benefits for infant sleep, respiration, excretion, colic reduction and growth improvement, especially in premature infants who require more nursing and support, touch may have special effects.^12^,^13^ However, more research is needed on the exact effect of baby touch on premature infants.

The present study aimed to explore the multifaceted effects of touch on premature infants. To ensure the accuracy and reliability of this study, a randomized case-control trial was conducted to comprehensively observe the effects of three months touch on the growth and development, immunological function, sleep quality, and emotional communication of premature infants. The results will provide a more scientific and powerful basis for the nursing of premature infants.

## METHODS

This was a retrospective study. A total of 64 premature infants with a gestational age of 30-36 weeks were selected at Maternity & Child Care Center of Qinhuangdao from June 2022 to May 2023, and randomly divided into experimental group (n=32) and control group (n=32) according to different nursing intervention methods. Data were retrieved from the hospital information and management system. Collect their various information of all patients. No significant differences were found in gender, gestational age, birth weight, birth length, or head circumference at birth between the two groups(*p>* 0.05), as seen in Tables-I and II.

**Table-I:** Comparison of basic information between the two groups (n = 64).

Group	Gender (male/female)	Gestational age
Control group	18/14	34.116 ± 1.400
Experimental group	17/15	34.147 ± 1.242
*χ^2^*/*t*	0.063	-0.094
*P*	0.802	0.925

**Table-II:** Comparison of growth and development indicators between the two groups (*χ̅*±*S*) (n = 64).

Group	On the 1st day			In the 3rd month		

	Body weight (kg)	Body length (cm)	Head circumference (cm)	Body weight (kg)	Body length (cm)	Head circumference (cm)
Control group	2.045 ± 0.214	45.308 ± 2.787	32.759 ± 2.445	6.225 ± 0.111	58.477 ± 1.771	36.977 ± 2.094
Experimental group	2.051 ± 0.218	45.200 ± 2.905	32.747 ± 2.339	6.456 ± 0.162	59.578 ± 1.876	38.107 ± 1.856
*t*	-0.110	0.153	0.020	-6.656	-2.415	-2.305
*P*	0.913	0.879	0.984	8.426E-9	0.019	0.025

### Ethical Approval:

The study was approved by the Institutional Ethics Committee of Maternity & Child Care Center of Qinhuangdao (No.: QHDFY-2023041910; date: April 19, 2023), and written informed consent was obtained from the participants’ guardians

### Inclusion criteria:[PJMS1]

- Birth at a gestational age between 30-36 weeks;
- Good general condition at birth, without severe complications or gastrointestinal abnormalities;
- Birth weight < 2.5 kg;
- One minute and five minutes Apgar scores > 8.

### Exclusion criteria

- A birth weight of < 1,000 g;
- Chromosomal disorders such as neonatal hypoxic-ischemic encephalopathy, neonatal respiratory distress syndrome, severe intrauterine organ dysfunction and genetic metabolic diseases, congenital malformations, and trisomy 21 syndrome.

The control group received routine comprehensive nursing care for newborns (maintaining stable body temperature, maintaining airway patency, effectively preventing infections, and providing scientific and balanced nutritional management). On this basis, the experimental group was additionally treated with touch intervention using the international standard touch method. Under a warm and peaceful indoor environment, touch was performed at an appropriate time, such as after bathing, before sleeping, etc. The caretakers needed to wash their hands and trim their nails. The newborns were placed on a touch table in the supine position, and touch was started from the head and face to the chest, abdomen, limbs, hands and feet, back, and then finally the buttocks. Each part was touched four to six times, twice a day, for 10-15 min each time. The touch action was gentle and coordinated with the infants’ emotions to achieve communication with the infants. When touching, the puerperants and family members were asked to participate in actively, and taught with touching techniques. After discharge, the infants were continuously touched by their mothers or family members. Touch was started on the 1^st^ day from birth.

### Observation indicators:

Growth and development indicators: The body length, body weight and head circumference of both the control group and the experimental group were measured and recorded on the 1^st^ day. In the 3^rd^ month, they were instructed to follow-up for measurement and recording.

### Immunological function indicators:

After obtaining the consent of the family members, blood was collected from newborns in both the control group and the experimental group on the 1st day and in the 3^rd^ month for the measurement of immunological function indicators such as CD^3+^, CD^4+^, CD^8+^, IgG, IgA and IgM.

### Sleep quality and Emotional communication indicators:

Both the control group and the experimental group were followed up and recorded in the 3^rd^ month.

### Statistical analysis:

Statistical calculations were conducted using SPSS 22.0. The *χ^2^* test was used for comparisons of binary variables (gender, etc.) between the groups. The continuous variables (body length, etc.) were expressed as (*χ̅*±*S*), and compared between the groups by the two independent samples *t*-test. *P*< 0.05 was considered as statistically significant.

## RESULTS

The results showed that the increases in body length and head circumference of the experimental group were significantly more obvious than those in the control group in the 3^rd^ month, with statistically significant differences (*p<* 0.05). The weight gain in the experimental group was significantly superior to the control group, with a statistically significant difference (*p<* 0.001), as shown in Table-II.

In the 3^rd^ month of intervention using the international standard touch method, the immunological function indicators (CD^3+^, CD^4+^, CD^8+^, IgG, IgA and IgM) in the experimental group were significantly higher than those in the control group, with statistical significance (*p<* 0.05), as shown in Table-III.

**Table-III:** Comparison of immunological function indicators between the two groups (*χ̅*±*S*) (n = 64).

Group	CD^3+^ (%)	CD^4+^ (%)	CD^8+^ (%)	IgG (g/L)	IgA (g/L)	IgM (g/L)
Control group	58.819 ± 5.361	37.787 ± 5.807	22.354 ± 3.671	8.639 ± 0.892	1.002 ± 0.107	0.825 ± 0.082
Experimental group	64.365 ± 5.831	42.050 ± 6.044	24.378 ± 2.865	9.494 ± 1.141	1.123 ± 0.161	0.885 ± 0.064
*t*	-3.960	-2.878	-2.459	-3.340	-3.557	-3.249
*P*	1.958E-4	0.005	0.017	0.001	0.001	0.002

In the 3rd month of touch intervention, the sleep time and sleep quality of the experimental group were both significantly superior to the control group, with statistically significant differences (*p<* 0.01), as presented in Table-IV.

**Table-IV:** Comparison of sleep quality indicators between the two groups (*χ̅*±*S*) (n = 64).

Group	Sleep time (h)	Sleep quality (%)
Control group	15.681 ± 0.890	84.266 ± 4.107
Experimental group	16.559 ± 0.898	87.734 ± 3.793
*t*	-3.929	-3.510
*P*	2.171E-4	0.001

The interaction time and intimacy with parents in the experimental group were significantly longer and higher than those in the control group in the 3rd month, with statistically significant differences (*p<* 0.05), as exhibited in Table-V.

**Table-V:** Comparison of emotional communication indicators between the two groups (*χ̅*±*S*) (n = 64).

Group	Interaction time (min)	Intimacy (%)
Control group	30.563 ± 3.860	87.438 ± 5.130
Experimental group	33.938 ± 3.528	90.438 ± 5.073
*t*	-3.651	-2.352
*P*	0.001	0.022

## DISCUSSION

In the present study, baby touch could significantly improve the growth and development indicators such as body length, body weight and head circumference of premature infants, and enhance their emotional communication abilities such as interaction time and intimacy. This is consistent with the report by Yoanita R et al.^14^ that baby touch can significantly improve body weight, growth and sleep while reducing hyperbilirubinemia of full-term infants, as well as significantly increase the weight gain of premature infants, reduce their response to pain, and increase their interaction with parents. In addition, our study also found that baby touch could significantly improve the levels of immunological function indicators such as CD^3+^, CD^4+^, CD^8+^, IgG, IgA and IgM, as well as enhance sleep time and quality. This may result from that touch can stimulate the skin and sensory system, promote the development of the nervous system, and enhance the immunological function of premature infants. Moreover, touch can also make premature infants feel safe and comfortable, thereby improving their sleep quality and enhancing their emotional communication with their parents.

According to WHO estimates^15^, 15 million babies worldwide are born prematurely at 37 weeks of gestation or less each year. Premature birth is the leading cause of death in children under five years old in the world. Extreme premature birth under 28 weeks of gestation increases the risk of future motor and cognitive impairments, including long-term neurological damage such as cerebral palsy, learning and behavioral difficulties, and respiratory problems such as bronchopulmonary dysplasia.^16^,^17^ It has been shown^18^ that appropriate therapeutic environments and nursing methods are beneficial for the rehabilitation and development of premature infants. Creating a healing environment and clustering nursing care can significantly improve respiration, heart rate, blood oxygen saturation and systolic blood pressure, while increasing sleep time and reducing wakefulness and pain score.

The WHO defines kangaroo care(KC) as a nursing method that allows skin-to-skin contact between premature infants or newborns and their parents, which can promote the health and well-being of infants and their families.^19^ KC helps improve body temperature control, promote breastfeeding, reduce stress, and promote the maturation of sleep-wake cycles, thereby facilitating to establish intimate relationships, reducing infection risk, and shortening hospital stays.^20^,^21^ In a previous study^22^, low birth-weight infants stable on respiratory support were treated with KC for one hour, and vital signs and ventilator parameters were recorded every 15 minutes before, during and after treatment. No significant changes were found in body temperature, respiratory rate or saturation, but HR and Fio2 were slightly higher during treatment than those before and after surgery.

### Limitations:

It includes small sample size and we did not conduct stratified analysis on premature infants with different gestational ages, body weights and disease conditions. In future research, the sample size can be further expanded, and the impacts of these factors on the touch effect will be considered. Additionally, the combined effect of touch and other nursing methods (such as music therapy, swimming, etc.) can also be studied to explore more effective nursing methods.

## CONCLUSIONS

Baby touch has a positive effect on premature infants by promoting their growth and development, strengthening their immunological function, improving their sleep quality, and enhancing their emotional communication abilities. Therefore, it is recommended to promote baby touch technology in clinical practice to help premature infants better adapt to the environment and grow. Moreover, parents can also provide touch at home to promote the physical and mental development of premature infants. It should be noted that during touch, attention should be paid to the intensity and time to avoid causing damage to premature infants. It is best to provide touch under the guidance of professional medical staff.

### Authors’ Contributions:

**QD and HZ** carried out the studies, data collection, drafted the manuscript, and are responsible and accountable for the accuracy or integrity of the work.

**MG, QY, HY and YM:** Performed the statistical analysis and participated in its design.

All authors read and approved the final manuscript and are responsible for the integrity of the study.
